# Supplementary material for: Non-hermiticity in spintronics: oscillation death in coupled spintronic nano-oscillators through emerging exceptional points
Source: Nat Commun. 2024 Feb 1;15:971. doi: 10.1038/s41467-023-44436-z (PMC10834588; doi:10.1038/s41467-023-44436-z)
Supplement: Supplementary file 1 — Supplementary Information [file 41467_2023_44436_MOESM1_ESM.pdf]

# – Supplementary information –
Non-hermiticity in spintronics: oscillation death in coupled spintronic nano-oscillators through emerging exceptional points

Steffen Wittrock,[1,2,*] Salvatore Perna,[3] Romain Lebrun,[1] Katia Ho,[1] Roberta Dutra,[4] Ricardo Ferreira,[5] Paolo Bortolotti,[1] Claudio Serpico,[3] and Vincent Cros[1,†]

[1]*Laboratoire Albert Fert, CNRS, Thales, Université Paris-Saclay, 1 Avenue Augustin Fresnel, 91767, Palaiseau, France*
[2]*Helmholtz-Zentrum Berlin für Materialien und Energie GmbH, Hahn-Meitner-Platz 1, 14109 Berlin, Germany*
[3]*Department of Electrical Engineering and ICT, University of Naples Federico II, 80125 Naples, Italy*
[4]*Centro Brasileiro de Pesquisas Fésicas (CBPF), Rua Dr. Xavier Sigaud 150, Rio de Janeiro 22290-180, Brazil*
[5]*International Iberian Nanotechnology Laboratory (INL), 471531 Braga, Portugal*
(Dated: December 12, 2023)

In this supplementary information, we elaborate on the used theoretical model and discuss the linear and nonlinear dynamics. We present simulations of the full nonlinear equations. Furthermore, we show supplementary experimental data.

S1. Theory

Thiele's equations. The model equations for the two coupled STVOs are the following [1]:

$$\frac{d\boldsymbol{\rho}_i}{dt} = \Omega_i(\rho_i, I_i)\boldsymbol{n}_i \times \boldsymbol{\rho}_i + \left[C_i I_i - d_i \cdot \Omega_i(\rho_i, I_i)\right]\boldsymbol{\rho}_i + K \cdot \boldsymbol{\rho_j}\,, \quad \text{(S1)}$$

where $i, j \in \{1, 2\}, i \neq j$, denoting STVO 1 or 2. The symbol $\boldsymbol{n}_i$ denotes the unit vector along the symmetry axis of the oscillator, I_i is the injected current, the variable $\boldsymbol{\rho}_i$ is the in-plane vortex core position normalized to the radius R_i of the device, Ω_i is the conservative oscillations angular frequency, d_i a dimensionless damping constant and C_i is a parameter determining the efficiency of the spin torque effect. This latter parameter determines also the critical current $I_{c,i}$ needed for exciting self-sustained oscillations in the uncoupled case. The angular frequency Ω_i is a function of I_i and of the magnitude $\rho_i = |\boldsymbol{\rho}_i|$: $\Omega(\boldsymbol{\rho}, I) = \Omega_{ms}(1/[1 - (\rho/2)^2]) + n_{oe}I(1 - \rho^2/2)$, where Ω_{ms} and n_{oe} take into account the influence on the frequency of the magnetostatic field generated by the magnetic vortex state and of the Oersted field generated by the injected current, respectively. The coupling between STVO i and j is described by a 2×2 matrix:

$$K \cdot \boldsymbol{\rho}_j = k_d \boldsymbol{\rho}_j + k_c \boldsymbol{n}_j \times \boldsymbol{\rho}_j.$$

where k_c and k_d are conservative and dissipative coupling coefficients, respectively.

$\mathcal{PT}$-symmetry. It is interesting to notice that at perfect gain compensation, $\beta_1 = -\beta_2 = \beta$, and the same frequencies of the uncoupled oscillators, $\omega_1 = \omega_2 = \omega$, the natural frequencies are given by the simplified formula: $\nu_{1,2} = \omega \pm \sqrt{k^2 - \beta^2}$. Thus, the natural frequencies are both real when $\beta < k$, while for $\beta > k$ an imaginary part appears. The system has in this case a $\mathcal{PT}$-symmetry when the frequencies are real and a broken $\mathcal{PT}$-symmetry when the frequencies are complex. The symmetry-breaking bifurcation occurs when $\beta = k$ and this condition corresponds to an EP. Representing an interesting case, this situation however requires a very fine tuning of the two oscillators' properties.

$\Omega_{ms,1}$ [MHz]	$2\pi \cdot 225$
$\Omega_{ms,1}$ [MHz]	$2\pi \cdot 233$
$n_{Oe,1} = n_{Oe,2}$ [MHz/mA]	$2\pi \cdot 3$
$d_1 = d_2$	0.1
C_1 [MHz/mA]	22.22
C_2 [MHz/mA]	20.18

Table S1: System parameters for the calculated eigenvalues.

System parameters. The used model parameters of the system, derived from the experimental data, are listed in table S1.

Exchange of eigenvectors. The oscillations in practice take place in the two oscillators. However, in the coupled system the dynamics must be rather understood as a collective phenomenon. In this respect, it is interesting to correlate the eigenvectors with their localization in each STVO.

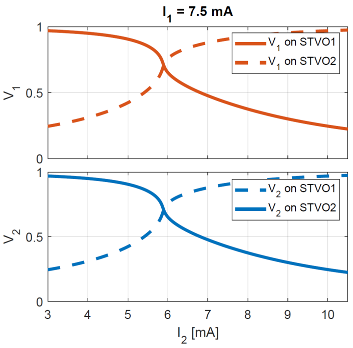

Figure S1: Projection of the system's eigenvectors onto the STVOs in order to determine their localization.

In fig. S1, we plot the projection of the two eigenvectors v_1 and v_2 on STVO 1 and STVO 2 at an exemplary current

* steffen.wittrock@helmholtz-berlin.de
† vincent.cros@cnrs-thales.fr

value of $I_1^* = 7.5\,\mathrm{mA}$ (see also fig. 4g). When the eigenvalues approach each other (fig. 4g), there is an exchange of the eigenvectors' components. In fig. S1, the eigenvector v_1 is localized mainly in STVO 1 and v_2 in STVO 2 for current values of $I_2 \lesssim 6\,\mathrm{mA}$. For $I_2 \gtrsim 6\,\mathrm{mA}$, the eigenvectors change their localization and v_1 (v_2) is majorly identified in STVO 2 (1).

S2. Nonlinear dynamics

The presented results have their theoretical foundation in the linearized theory of the coupled system dynamics although the measured spectra refer to phenomena (e.g self-oscillation regimes) which can only be rigorously explained with nonlinear theory arguments. It is therefore important to show the consistency of the linear model with computations of nonlinear dynamics of the coupled STVOs.

Numerical model. Based on the single STVO measured spectra (shown in fig. S3 further below), the parameters of the Thiele equation for each oscillator have been determined. In particular, from the critical current $I_{c,i}$ and the corresponding self-oscillation frequency, the value of the constants $\Omega_{ms,i}$, C_i assuming certain values for n_{Oe} and d_i can be estimated. The nonlinear current dependence of the measured self-oscillation frequency is reproduced by assuming a polynomial dependence on the vortex core position of the damping term $d_i \to d_i(\rho_i)$. We remark that this modeling procedure is for the single STVO uncoupled from the other one. The coupling effect is determined as described in the section *Experimental emergence of EPs* of the main text.

Simulations. In fig. S2, the computed spectra from time integration of the nonlinear dynamics of the coupled STVOs (eq. (S1)) are shown. Fixing the current I_1, the dynamics of the coupled oscillators is simulated for each value of the current I_2 in the range $(3, 10.5)$ mA, taking an ensemble of N initial conditions randomly distributed in a disk around the orgin of radius $\rho/R = 0.01$. Then from the self-oscillations regime we estimate the power as: $P = 1/N\ \log_{10}(\sum_i |\tilde{x}_{1,i} + \tilde{x}_{2,i}|^2)$, where $\tilde{x} = FFT[x]$, and the notation $x_{n,i}$ refers to the x coordinate of the vortex core of the STVO-n.

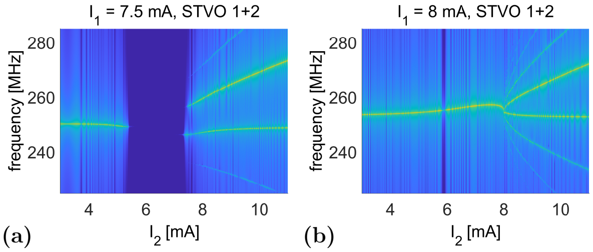

Figure S2: Frequency spectra computed by integrating the nonlinear model of the coupled STVOs for two current I_1^* values. The color scale is in arbitrary units and is analogous to figures which show the measured spectra.

The spectra computed from the nonlinear model confirm the validity of the approach used in the Experiments section to estimate the coupling constant k_{EP}. The similarity of fig. 3a and fig. S2b is a strong indication of the fact that the branching of the frequency in the measured spectra is due to the passage in the proximity of an EP. The agreement between experimental results and numerical simulations in terms of frequency value and frequency gap when the spectra become double peaked is obtained also for the other cases where the current I_1^* is changed in the range $[6.9; 8]$ mA. Note that also modulation peaks are visible (such as also present experimentally, e.g., in fig. 4d), however unrelated to non-hermitian physics discussed here. Finally, the agreement on the amplitude death current ranges is not surprising since the use of the linear theory for their estimation is a rigorous result of the nonlinear system theory.

S3. Experimental description

Exact fabrication of the spintronic oscillator devices is detailed in the Methods section of the main text. Here, we provide additional experimental information and show and discuss additional data.

Working principle and measurements. Measurements are conducted under an applied out-of-plane field of $\mu_0 H_\perp = 360\,\mathrm{mT}$. It tilts the in-plane magnetization of the SAF slightly into the perpendicular direction. That induces an out-of-plane spin current polarization, necessary for an efficient spin transfer torque (STT) [2]. The STT provides the gain mechanism in the STVOs and is generated by injected dc currents, which are separately controlled for the two STVOs by two dc current sources. The TMR effect converts the vortex magnetization dynamics into an electrical rf signal. The electrical rf output signal of each oscillator is amplified by 30 dB and injected into the field line located above the other STVO (see fig. 1) in order to implement a symmetric coupling scheme through the generated rf Oersted fields. The coupling is nonlocal and the STVOs are in our case several millimeters apart from each other (however, their distance can be arbitrarily chosen). In the electrical circuit, the dc and rf current parts are separated through a bias tee and the dc electrical properties are monitored by a voltmeter. The emitted rf signals of both coupled STVOs are combined and recorded by a spectrum analyzer.

Symmetry of the coupled STVO system. In order to reveal the emergence of an exceptional point and complex dynamics in the coupled STVO system, the uncoupled characteristics of the oscillators should be sufficiently similar for approximately realizing a symmetric situation with reciprocal coupling.

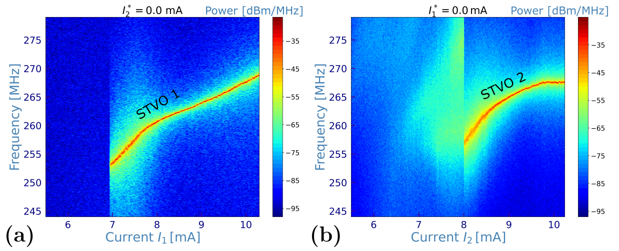

Figure S3: Frequency spectra of the two independent STVOs.

In fig. S3, the power spectra for the two uncoupled STVOs are measured separately. They show that in the vicinity of the threshold current for self-oscillations the frequency characteristics of the two STVOs are very similar. The region close to the onset of oscillations is the one of interest for the study of EPs.

However, the realization of a perfectly symmetric system

is experimentally difficult owing to small deviations in the complex, nanometer scale fabrication process, and the coupled system is very sensitive to small parameter changes in the vicinity of the EP. Performing measurements at constant current I_2^* while changing the current I_1 in STVO 1 (fig. S4) provides qualitatively different results than the ones shown beforehand fixing the current in STVO 1. We observe a nontrivial behavior of the coupled system with either suppressed or stabilized oscillations of the STVOs. At $I_2^* = 7.95\,\mathrm{mA}$ (fig. S4a), STVO 2 is not sustained, but the critical current of STVO 1 slightly shifted to a larger current (compare with fig. S3a). At $I_2^* = 8\,\mathrm{mA}$ (fig. S4b), corresponding to the critical current of STVO 2 (see fig. S3b), the latter's auto-oscillations are suppressed for $I_1 \lesssim 6\,\mathrm{mA}$, and are subject to stochastic effects above 6 mA until the oscillations of STVO 1 become stabilized. For $I_2^* = 8.05\,\mathrm{mA}$ and $I_2^* = 8.1\,\mathrm{mA}$ (figs. S4c & S4d, resp.), we observe the stabilization of STVO 2's auto-oscillations at smaller currents I_1 and a suppression of STVO 2's oscillations after its frequency has crossed that of STVO 1 at $I_1 \gtrsim 8.5\,\mathrm{mA}$.

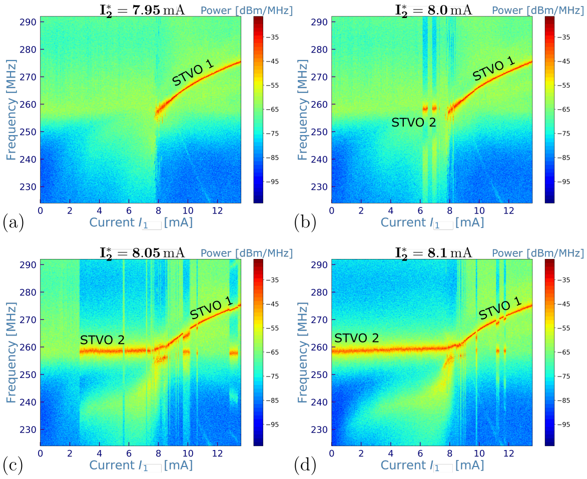

Figure S4: Measured frequency spectra of the coupled system vs. current I_1 for different constant currents I_2^*.

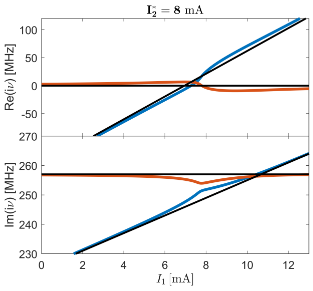

Figure S5: Theoretically modeled eigenvalues vs. current I_1 for constant current $I_2^* = 8\,\mathrm{mA}$. All parameters are chosen identical as the ones at constant currents I_1^* shown above and discussed in the main text. The coupling phase is set to $\phi_k = 10\,^\circ$.

In fig. S5, we show that also here the linearized theory qualitatively reproduces the observed features, employing the same parameters as for the inverse case presented in the main text, and a coupling phase of $\phi_k = 10^\circ$. Note that in this case, an extreme fine tuning of the theoretically applied parameters would be necessary in order to thoroughly reproduce the experimental characteristics since already a very subtle change of I_2^* in the vicinity of the EP significantly changes the oscillation properties. Hence, here we choose $I_2^* = 8\,\mathrm{mA}$ as a representative current value. It can be observed that $\mathrm{Re}(i\nu_2)$ (red-curve) is close to the zero stability axis. Increasing I_1 from 0 first increases $\mathrm{Re}(i\nu_2)$ leading to possible stabilized oscillations up to 8 mA, such as reflected experimentally in figs. S4b - S4d. A larger current I_2^* stabilizes the oscillations already at lower I_1 while a smaller one leads to a shift of $\mathrm{Re}(i\nu_2)$ entirely below the zero axis, also reflected experimentally in fig. S4. For current values of $I_1 \gtrsim 8\,\mathrm{mA}$, $\mathrm{Re}(i\nu_2)$ (red-curve) becomes negative while staying close to the stability axis. This leads to oscillation death in this regime while partly seeing oscillations when thermally induced transitions into the positive plane occur (see fig.S4c - S4d). The value I_1 at which the blue curve in fig. S5 crosses the zero axis is slightly larger than at zero coupling (black curve), reproducing the observed larger critical current at $I_2^* = 7.95\,\mathrm{mA}$ in fig. S4a.

The measurements emphasize the physical richness of the coupled system even at only little asymmetry between the two oscillators. It can be stated that the individual parameters of the two STVOs are of critical importance, especially when being close to an EP. Furthermore, the nature of the coupling between the two oscillators is responsible for the observed characteristics and leads to manifold interesting effects in the collective system and hence, will be an additional important control parameter in future experiments.

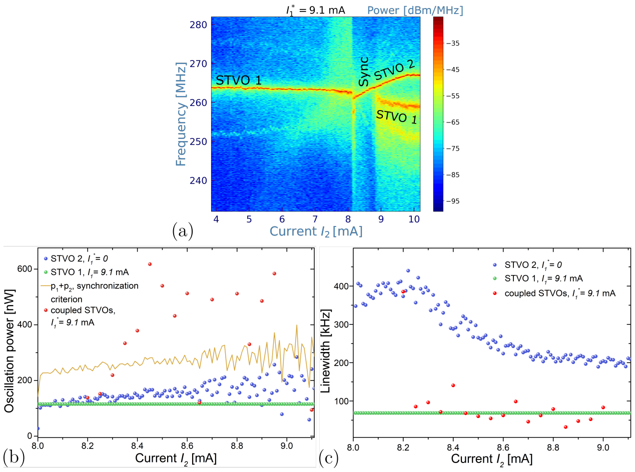

Figure S6: Experimental characterization in the regime of mutual synchronization of the two coupled STVOs: $I_1^* = 9.1\,\mathrm{mA}$, $\mu_0 H_\perp = 360\,\mathrm{mT}$, $T = 300\,\mathrm{K}$. (a) Measured spectra. (b-c) Oscillation power and linewidth evaluated for the synchronized and the uncoupled case.

Synchronization at larger current densities. In fig. S6a, we show the power spectra recorded for larger current densities I_1^* in our experiment. We observe the well-known, intrinsically nonlinear effect of mutual synchronization between $I_2 = 8.2$ and $8.95\,\mathrm{mA}$. Such synchronization implies an increase of the emitted power and a decrease of the spectral linewidth as shown in figs. S6b-c. The dark-yellow line in fig. S6b defines the mutual synchronization criterion [3] beyond which the synchronized power indicates efficient synchronization. Note that the mutual synchronization phe-

nomenon is the best-explored in the nonlinear coupled dynamics of STNOs and it is noteworthy that here, it co-exists with the described complex dynamics at lower current densities linked to non-hermitian physics, with the only practical difference in the supplying current. However, we want to emphasize that mutual synchronization in this context must not be necessarily taken for granted, but, depending on the system parameters, such as especially the type and strength of the coupling, different complex situations might occur (chaos, bistability, etc.), and need to be explored.

[1] P. Bortolotti, E. Grimaldi, A. Dussaux, J. Grollier, V. Cros, C. Serpico, K. Yakushiji, A. Fukushima, H. Kubota, R. Matsumoto, and S. Yuasa, Parametric excitation of magnetic vortex gyrations in spin-torque nano-oscillators, Physical Review B **88**, 10.1103/physrevb.88.174417 (2013).

[2] A. Dussaux, B. Georges, J. Grollier, V. Cros, A. Khvalkovskiy, A. Fukushima, M. Konoto, H. Kubota, K. Yakushiji, S. Yuasa, K. Zvezdin, K. Ando, and A. Fert, Large microwave generation from current-driven magnetic vortex oscillators in magnetic tunnel junctions, Nature Communications **1**, 1 (2010).

[3] A. Slavin and V. Tiberkevich, Nonlinear auto-oscillator theory of microwave generation by spin-polarized current, IEEE Transactions on Magnetics **45**, 1875 (2009).

[4] S. Wittrock, S. Perna, R. Lebrun, K. Ho, R. Dutra, R. Ferreira, P. Bortolotti, C. Serpico, and V. Cros, Non-hermiticity in spintronics: Oscillation death in coupled spintronic nano-oscillators through emerging exceptional points – Raw Data (2023).
